# tBRD-1 and tBRD-2 regulate expression of genes necessary for spermatid differentiation

**DOI:** 10.1242/bio.022467

**Published:** 2017-02-24

**Authors:** Ina Theofel, Marek Bartkuhn, Thomas Boettger, Stefanie M. K. Gärtner, Judith Kreher, Alexander Brehm, Christina Rathke

**Affiliations:** 1 Philipps-Universität Marburg, Department of Biology, Marburg 35043, Germany; 2 Institute for Genetics, Justus-Liebig-Universität, Giessen 35392, Germany; 3 Department of Cardiac Development and Remodeling, Max Planck Institute for Heart and Lung Research, Bad Nauheim 61231, Germany; 4 Philipps-Universität Marburg, Institute of Molecular Biology and Tumor Research, Marburg 35037, Germany

**Keywords:** Testis-specific transcription, tTAFs, tMAC, Mediator complex, BET proteins

## Abstract

Male germ cell differentiation proceeds to a large extent in the absence of active gene transcription. In *Drosophila*, hundreds of genes whose proteins are required during post-meiotic spermatid differentiation (spermiogenesis) are transcribed in primary spermatocytes. Transcription of these genes depends on the sequential action of the testis meiotic arrest complex (tMAC), Mediator complex, and testis-specific TFIID (tTFIID) complex. How the action of these protein complexes is coordinated and which other factors are involved in the regulation of transcription in spermatocytes is not well understood. Here, we show that the bromodomain proteins tBRD-1 and tBRD-2 regulate gene expression in primary spermatocytes and share a subset of target genes. The function of tBRD-1 was essential for the sub-cellular localization of endogenous tBRD-2 but dispensable for its protein stability. Our comparison of different microarray data sets showed that in primary spermatocytes, the expression of a defined number of genes depends on the function of the bromodomain proteins tBRD-1 and tBRD-2, the tMAC component Aly, the Mediator component Med22, and the tTAF Sa.

## Abstract (precis)

**Summary:** Testis transcriptome analyses indicate that a defined set of testis-expressed genes is regulated by the testis-specific bromodomain proteins tBRD-1 and tBRD-2 and members of the tMAC, Mediator, and tTFIID complexes.

## INTRODUCTION

In *Drosophila melanogaster* and mammals, the post-meiotic phase of spermatogenesis (spermiogenesis) is characterized by extensive morphological cell changes (Rathke et al., 2014). In flies, transcription almost ceases as the cells enter meiotic division; therefore, these changes mainly rely on proteins arising from translationally repressed and stored mRNAs synthesized in primary spermatocytes (Olivieri and Olivieri, 1965; White-Cooper et al., 1998). Hence, a tightly regulated gene transcription program is required to ensure proper spermiogenesis and male fertility.

In primary spermatocytes, numerous transcripts are synthesized and translationally repressed (Fuller, 1993; White-Cooper et al., 1998). Transcription of the corresponding genes (spermiogenesis-relevant genes) depends on two testis-specific transcription complexes: the testis meiotic arrest complex (tMAC), and the testis-specific TFIID complex, which consists of testis-specific TATA box binding protein-associated factors (tTAFs) (Beall et al., 2007; Hiller et al., 2004, 2001). Recruitment of tTAFs to chromatin requires the coactivator complex Mediator, and localization of Mediator subunits to chromatin depends on tMAC (Lu and Fuller, 2015). Based on these data, it has been suggested that Mediator acts as a key factor in a tTAF- and tMAC-dependent gene regulatory cascade that leads to transcriptional activation of spermiogenesis-relevant genes (Lu and Fuller, 2015).

Acetylated lysines of histone play an important role in gene transcription (Sanchez and Zhou, 2009). These histone modifications are recognized by bromodomain-containing proteins (Dhalluin et al., 1999). The bromodomain forms a well-conserved structure within functionally distinct proteins, such as histone acetyltransferases, chromatin-remodeling factors, transcriptional co-activators and mediators, and members of the bromodomain and extra-terminal (BET) family (Josling et al., 2012). Members of the BET family are characterized by having one (in plants) or two (in animals) N-terminal bromodomains and a conserved extra-terminal domain that is necessary for protein–protein interactions (Florence and Faller, 2001; Matangkasombut et al., 2000; Platt et al., 1999). BET proteins contribute to transcription mainly by recruiting protein complexes, e.g. transcription factors and chromatin remodelers (Josling et al., 2012; Krogan et al., 2003; Matangkasombut et al., 2000). In mammals, the BET proteins BRD2, BRD3, BRD4, and BRDT are expressed in male germ cells (Klaus et al., 2016; Shang et al., 2004). BRDT is involved in gene expression during spermatogenesis, among other roles (Berkovits et al., 2012; Gaucher et al., 2012), but the functions of BRD2, BRD3, and BRD4 in male germ cells are not well understood.

In *Drosophila*, three testis-specific bromodomain proteins (tBRDs) have been described (Leser et al., 2012; Theofel et al., 2014). tBRD-1 contains two bromodomains, is essential for male fertility, and partially co-localizes with tTAFs in primary spermatocytes (Leser et al., 2012). Likewise, the BET family members tBRD-2 and tBRD-3 partially co-localize with tBRD-1 and tTAFs in primary spermatocytes (Theofel et al., 2014). In addition, subcellular localization of the three tBRDs depends on both tTAF function and the level of acetylation within the cell (Leser et al., 2012; Theofel et al., 2014). Loss of tBRD-1 function leads to an altered distribution of tBRD-2 and tBRD-3 and to a significant down-regulation of a subset of tTAF target genes (Theofel et al., 2014). Protein–protein interaction studies have revealed that tBRD-1 forms homodimers and also heterodimers with tBRD-2, tBRD-3, and tTAFs (Theofel et al., 2014). The loss of tBRD-1 or tBRD-2 leads to similar post-meiotic phenotypes, e.g. nuclear elongation defects (Kimura and Loppin, 2015; Leser et al., 2012). It has been postulated that in primary spermatocytes, tBRDs cooperate with tTAFs to regulate expression of selected spermiogenesis-relevant genes (Theofel et al., 2014).

Here, we show that a *tbrd-1-eGFP* transgene restores not only male fertility of *tbrd-1* mutants but also localization of tBRD-2 to chromosomal regions. Protein–protein interaction studies demonstrated that both bromodomains are dispensable for tBRD-1 homodimer formation and that the extra-terminal domain of tBRD-2 interacts with the C-terminal region of tBRD-1. Peptide pull-down experiments indicated that tBRD-1 but not tBRD-2 preferentially recognizes acetylated histones H3 and H4. Microarray analyses revealed that several genes are significantly down-regulated in *tbrd-2*-deficient testes. A comparison of different microarray data sets demonstrated that tBRD-1, tBRD-2, the tMAC component Aly, the Mediator component Med22, and the tTAF Sa share a subset of target genes. Finally, immunofluorescence stainings showed that the sub-cellular localization of tBRD-1 and tBRD-2 requires Aly function.

## RESULTS

### Expression of tBRD-1-eGFP reconstitutes proper sub-cellular localization of tBRD-2 in *tbrd-1* mutant spermatocytes

**Fig. 1.**
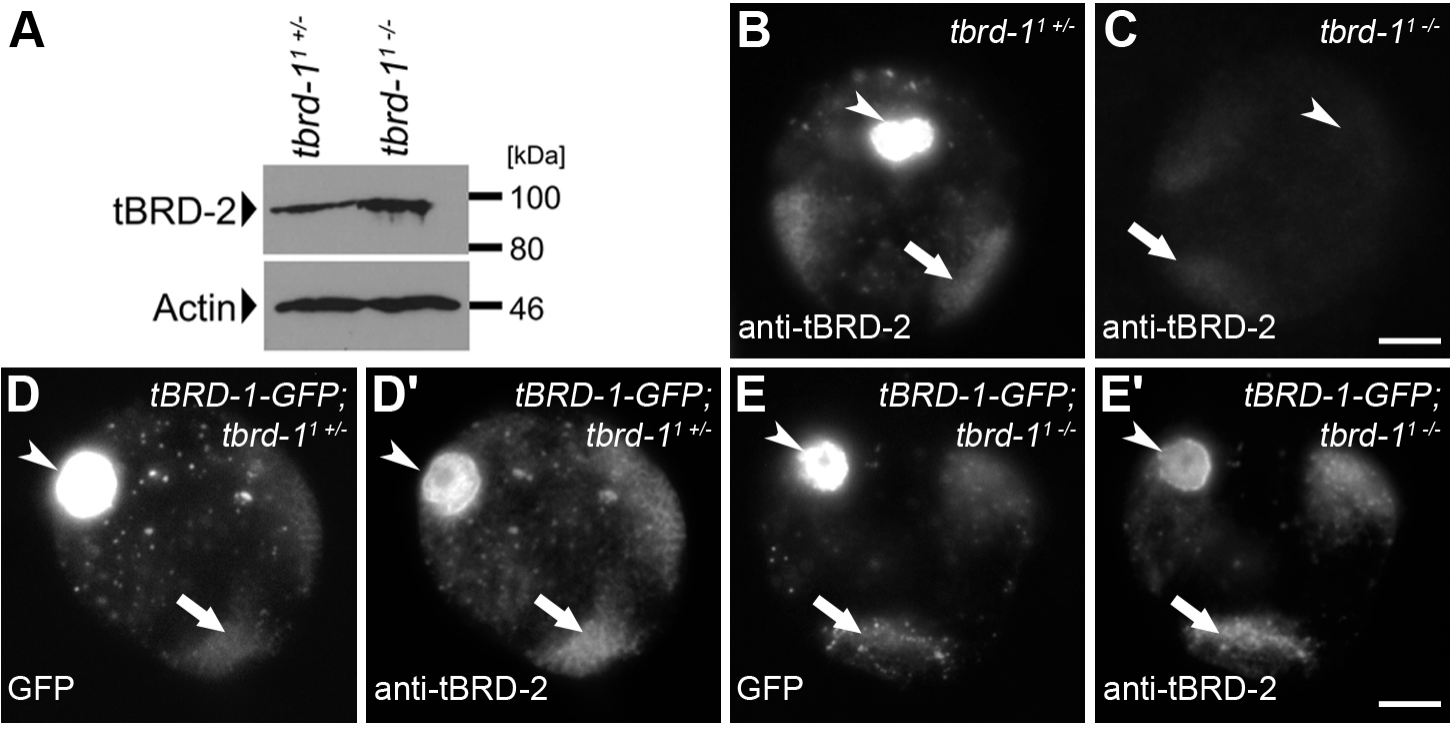
**The tBRD-1-eGFP fusion protein restores the localization of tBRD-2 to chromosomes in spermatocytes of *tbrd-1* mutants.** (A) Detection of tBRD-2 in heterozygous and homozygous *tbrd-1* mutant testes using anti-tBRD-2 antibody. Actin detected with anti-Actin antibody served as the control. (B,C) Single primary spermatocyte nuclei of (B) heterozygous and (C) homozygous *tbrd-1* mutants stained with an antibody against tBRD-2. (D–E′) Anti-tBRD-2 antibody staining of single primary spermatocyte nuclei of (D,D’) heterozygous and (E,E′) homozygous *tbrd-1* mutants expressing a tBRD-1-eGFP fusion protein. Arrows, chromosomal regions; arrowheads, nucleolus. Scale bars: 5 µm.

Recently, we have shown that the *tbrd-1* mutant phenotype is rescued by a *tbrd-1-eGFP* transgene, which contains the *tbrd-1* open reading frame together with 531 bp upstream of the translational start fused in frame with eGFP. The corresponding tBRD-1-eGFP fusion protein shows the same distribution as endogenous tBRD-1 (Leser et al., 2012). In addition, we have shown that tBRD-1 co-localizes with tBRD-2-eGFP, whose transgene contains the *tbrd-2* open reading frame and 591 bp upstream of the translational start fused in frame with eGFP. Furthermore, tBRD-1 function is required for proper tBRD-2-eGFP localization, and tBRD-1 interacts with tBRD-2-eGFP *in vivo* (Theofel et al., 2014). We have not been able to address whether localization of endogenous tBRD-2 protein is also dependent on tBRD-1 function. Towards this end, we raised a peptide antibody against tBRD-2 and tested its specificity in immunofluorescent stainings of *tbrd-2* knockdown and control testes (Fig. S1). Flies carrying a *UAS-tbrd-2^RNAi^* transgene were crossed with a *bam-*Gal4 driver line (*bam*≫*tbrd-2^RNAi^*) to down-regulate expression of tBRD-2 in the testis by RNAi. tBRD-2 was detected in spermatocyte nuclei of control testes (Fig. S1A), but almost no signal was observed in *tbrd-2* knockdown testes (Fig. S1B). We then analyzed the localization of endogenous tBRD-2 in heterozygous and homozygous *tbrd-1* mutants and in heterozygous and homozygous *tbrd-1* mutants expressing a tBRD-1-eGFP fusion protein (Fig. 1). Western blot analyses revealed that endogenous tBRD-2 levels were not reduced in *tbrd-1* mutant testes (Fig. 1A). In heterozygous *tbrd-1* mutant spermatocyte nuclei, endogenous tBRD-2 localized to chromosomal regions, nucleolus, and nuclear speckles in the nucleoplasm (Fig. 1B). However, although tBRD-2 protein levels were not reduced in homozygous *tbrd-1* mutant testes, only a faint tBRD-2 signal was visible in spermatocyte nuclei of homozygous *tbrd-1* mutants (Fig. 1C). By contrast, expression of a full-length tBRD-1-eGFP fusion protein in the homozygous *tbrd-1* mutant background reconstituted tBRD-2 localization to both the chromosomal regions and nucleolus (Fig. 1E′). These results extend our previous analysis and strengthen the idea that endogenous tBRD-1 and tBRD-2 interact and that tBRD-2 requires tBRD-1 for proper sub-cellular localization.

### The bromodomains of tBRD-1 are dispensable for homodimer formation, and the very C-terminus of tBRD-1 interacts with the extra-terminal domain of tBRD-2

**Fig. 2.**
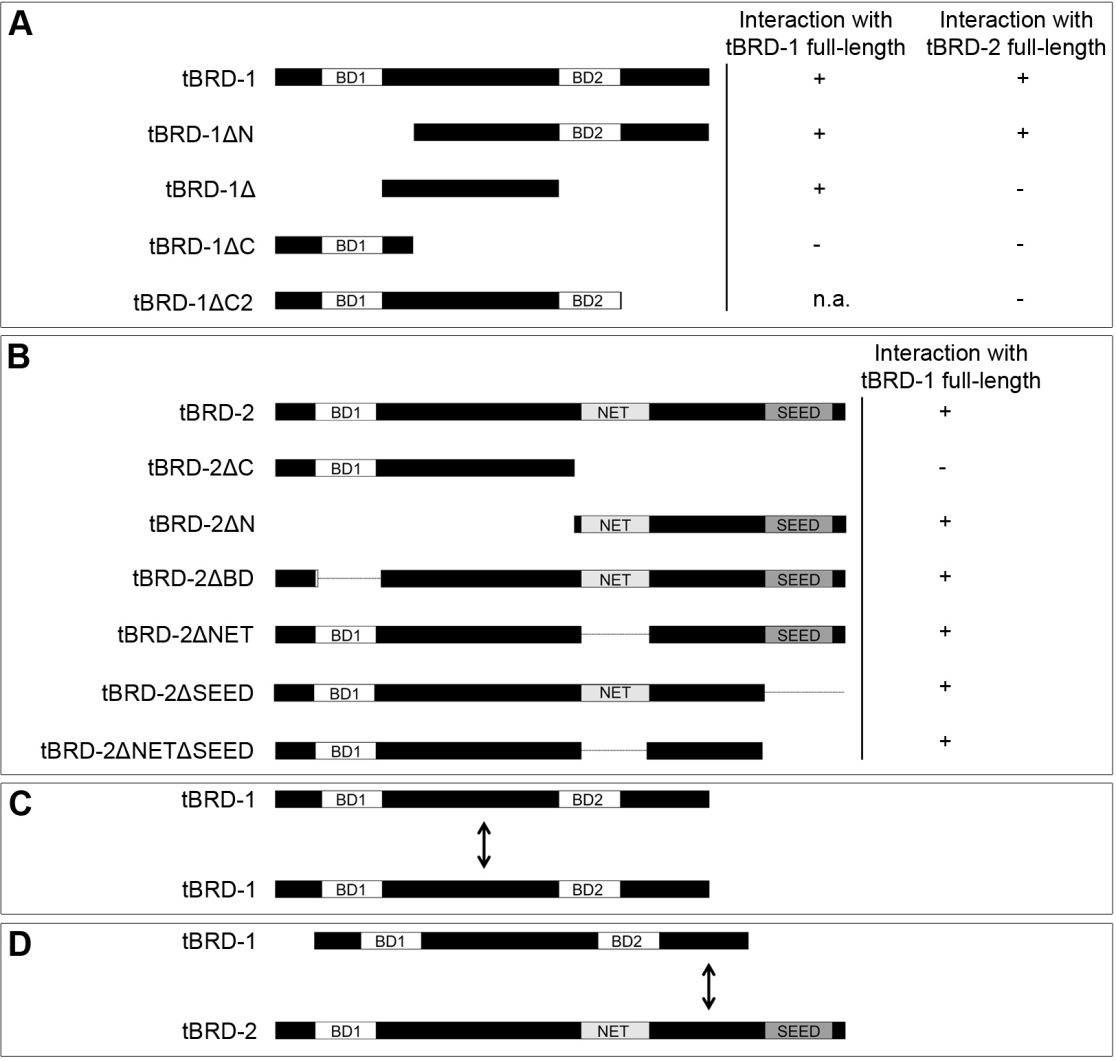
**The region between the two bromodomains is required for tBRD-1 homodimerization, and the C-terminus interacts with the extra-terminal domain of tBRD-2.** (A,B) Schematic overview of the full-length and mutated (A) tBRD-1 and (B) tBRD-2 proteins analyzed in yeast two-hybrid experiments for interaction with (A,B) full-length tBRD-1 and (A) tBRD-2 proteins. +, interaction observed; −, no interaction observed. (C) Schematic representation of the observed interaction between tBRD-1 and tBRD-1 via the region between the two bromodomains. (D) Schematic representation of the observed interaction between the C-terminus of tBRD-1 with the extra-terminal domain of tBRD-2. BD, bromodomain; NET, NET domain; SEED, SEED domain.

Recently, we have shown that tBRD-1 forms homodimers and also heterodimers with tBRD-2 (Theofel et al., 2014). Here, we aimed at mapping the interaction domains required for dimerization using a series of tBRD-1 and tBRD-2 truncation mutants in the yeast two-hybrid assay (Fig. 2; Figs S2 and S3). tBRD-1 and tBRD-2 contain several conserved domains, namely the bromodomains and an extra-terminal domain, which consists of a NET domain and a SEED domain and is predicted to mediate protein–protein interactions (Florence and Faller, 2001; Matangkasombut et al., 2000; Platt et al., 1999). Accordingly, we focused our analysis on these domains. Full-length tBRD-1 formed homodimers with tBRD-1ΔN, which lacks the first bromodomain (BD1) (Fig. 2A; Fig. S2B) and with tBRD-1Δ, which lacks both bromodomains and consists only of the spacer region that connects these two domains (Fig. 2A; Fig. S2B). No interaction was observed between full-length tBRD-1 and tBRD-1ΔC, which contains the first bromodomain but an incomplete spacer region (Fig. 2A; Fig. S2B). These results indicated that the spacer region between the bromodomains (amino acids 165–336) is essential for tBRD-1 homodimer formation (Fig. 2C). Next, we sought to determine which tBRD-2 sequences mediate binding to tBRD-1. We analyzed the interaction of several tBRD-2 deletion mutants with full-length tBRD-1 (Fig. 2B; Fig. S3A-D,F,H). We first mapped the binding to a C-terminal region containing the NET and SEED domains. Further analysis revealed that neither of these two domains was essential for tBRD-1 binding. Instead, tBRD-1 interaction required the region connecting the NET and SEED domains (amino acids 444–580). Finally, we showed that the C-terminus (amino acids 410–514) of tBRD-1 is required for heterodimerization with tBRD-2 (Fig. 2A; Fig. S3E,G). In summary, our results showed that the spacer region between the two bromodomains mediates tBRD-1 homodimerization (Fig. 2C) and indicated that tBRD-1 and tBRD-2 interact via the C-terminus of tBRD-1 and the region between the NET and the SEED domains of tBRD-2 (Fig. 2D).

### tBRD-1 recognizes acetylated histones H3 and H4 *in vitro*

**Fig. 3.**
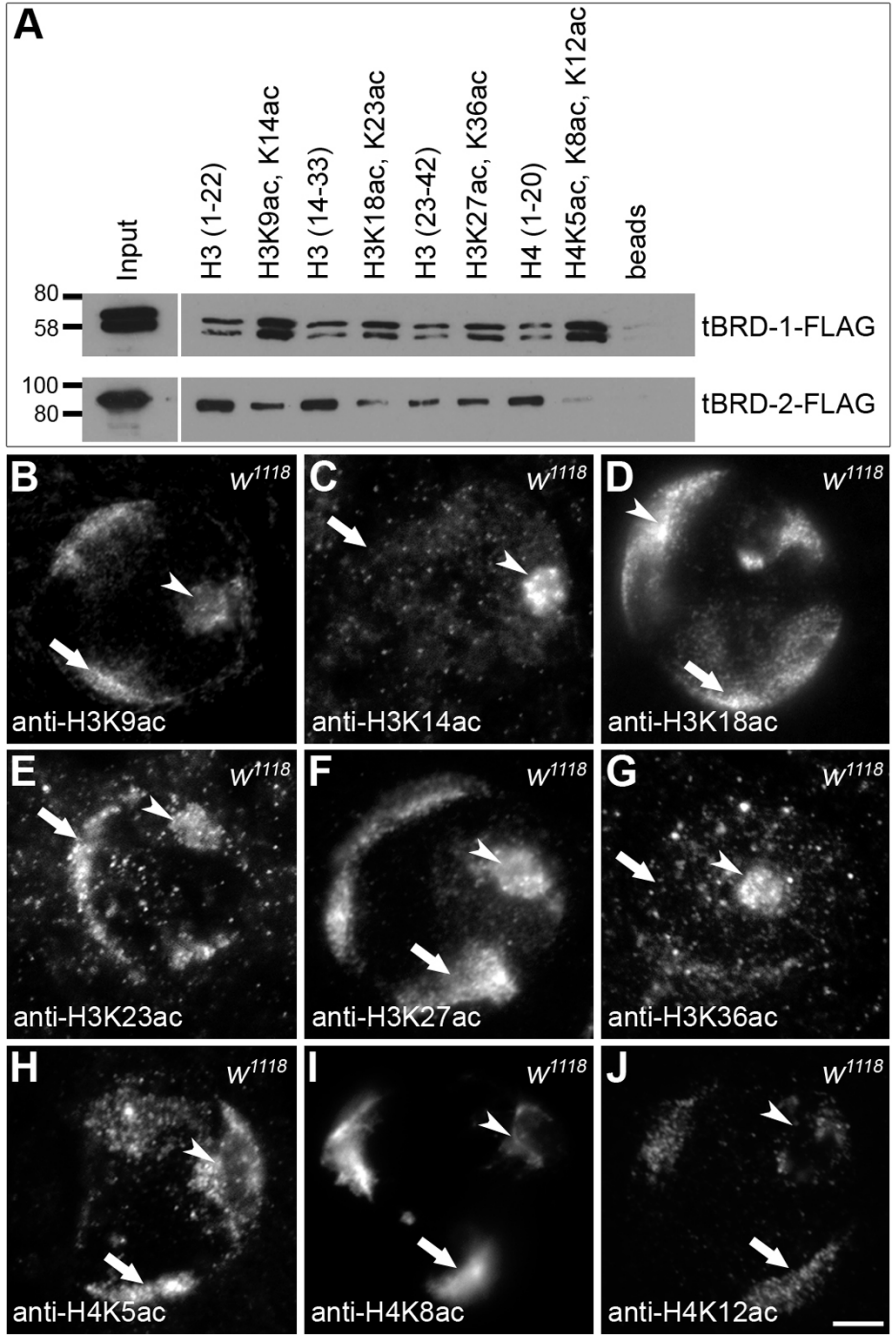
**tBRD-1 associates with acetylated histones.** (A) Association of recombinant tBRD-1-Flag and tBRD-2-Flag proteins with non-acetylated and acetylated histone H3 and H4 peptides [H3(1–22), H3(14–33), H3(23–42), H4(1–20)] was examined in peptide pull-down assays. Bound proteins were analyzed in western blots using tBRD-1- or tBRD-2-specific antibodies. Acetylated proteins are indicated, e.g. H3K9ac, K14ac: H3 acetylated on Lys9 and Lys14. (B-J) Single primary spermatocyte nuclei of *w^1118^* flies stained with specific antibodies against histone H3 acetylated on (B) Lys9, (C) Lys14, (D) Lys18, (E) Lys23, (F) Lys27, and (G) Lys36 or against histone H4 acetylated on (H) Lys5, (I) Lys8, and (J) Lys12. Arrows, chromosomes; arrowheads, nucleoli. Scale bar: 5 µm.

Previously, we have shown that localization of tBRD-1 and tBRD-2 to the chromosomal regions in spermatocytes is acetylation dependent (Leser et al., 2012; Theofel et al., 2014). This finding implied that tBRD-1 and tBRD-2 might directly interact with acetylated histone tails. To test this hypothesis, we purified recombinant tBRD-1 and tBRD-2 using the baculovirus system and performed peptide pull-down assays with histone H3 and histone H4 peptides that were unmodified or acetylated at specific residues. Immobilized peptides were incubated with recombinant tBRD-1 or tBRD-2, and bound proteins were analyzed in western blots using tBRD-1- or tBRD-2-specific antibodies (Fig. 3A). tBRD-1 bound to all unmodified or acetylated histone H3 and H4 peptides analyzed, in keeping with the idea that histone interactions might contribute to chromatin binding of tBRD-1, but tBRD-1 preferentially bound to acetylated histone tails (Fig. 3A). Likewise, tBRD-2 bound to all unmodified or acetylated histone peptides tested. In contrast to tBRD-1, however, tBRD-2 did not preferentially bind acetylated peptides, and acetylation instead appeared to reduce binding affinity. We concluded that tBRD-1 and tBRD-2 both interact with histone tails *in vitro* and that this binding reaction is sensitive to histone acetylation. To investigate whether these acetylated histones are present in spermatocytes, we stained them with immunofluorescent antibodies raised against different histone H3 and H4 acetylation marks (Fig. 3B–J). H3K9ac (Fig. 3B), H3K18ac (Fig. 3D), H3K23ac (Fig. 3E), H3K27ac (Fig. 3F), H4K5ac (Fig. 3H), H4K8ac (Fig. 3I), and H4K12ac (Fig. 3J) signals were detected at the chromosomal regions in primary spermatocytes (arrows) and acetylated histones H3K14ac and H3K36ac were barely detected at the chromosomal regions in primary spermatocytes (Fig. 3C,G, arrows).

### tBRD-2 and tBRD-1 share a subset of target genes

**Fig. 4.**
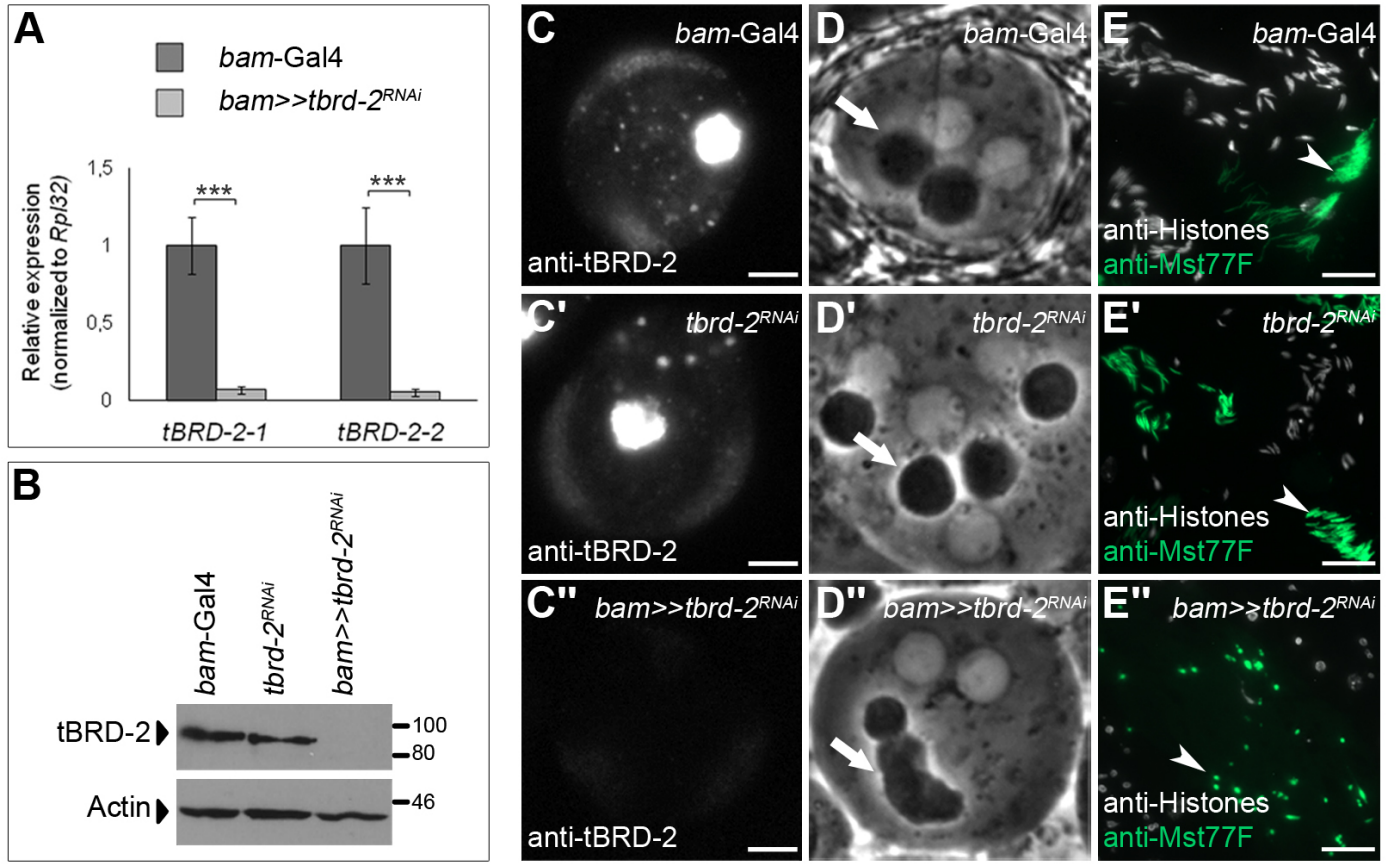
**RNAi-mediated knockdown of *tbrd-2* leads to defects in post-meiotic male germ cells.** (A) qPCR using cDNA from *tbrd-2* knockdown testes (*bam*≫*tbrd-2^RNAi^*) compared to control testes (*bam*-Gal4). Two different *tbrd-2*-specific primer pairs (tBRD-2-1 and tBRD-2-2) were used to detect *tbrd-2* transcript levels. The values were normalized to the mRNA expression level of *Rpl32*. Three technical replicates were performed. One-way ANOVA was used to evaluate statistical significance. *P*-values for significance: ***P≤0.001 (post hoc Tukey's honest significant difference test). (B) Western blot analysis of tBRD-2 proteins in *bam*≫*tbrd-2^RNAi^* testes, and in control testes (*bam*-Gal4 and *tbrd-2^RNAi^*). The detection of Actin with anti-Actin antibodies served as a loading control. (C–C″) Single primary spermatocyte nuclei of (C) *bam*-Gal4, (C′) *tbrd-2^RNAi^*, and (C″) *bam*≫*tbrd-2^RNAi^* flies stained with anti-tBRD-2 antibody. Photos had the same exposure time. Scale bar: 5 µm. (D–D″) Phase-contrast images of (D) control *bam*-Gal4, (D′) control *tbrd-2^RNAi^*, and (D″) *bam*≫*tbrd-2^RNAi^* early round spermatids. Arrows, Nebenkerne. (E–E″) Replacement of histones by Mst77F in post-meiotic spermatid nuclei of (E) *bam*-Gal4, (E′) *tbrd-2^RNAi^*, and (E″) *bam*≫*tbrd-2^RNAi^* visualized by immunofluorescence staining using antibodies against histones (white) and Mst77F (green). Arrowheads: E,E′, needle-like structure of mature sperm nuclei; E″, unelongated, round sperm nuclei. Scale bars: 20 µm.

In microarray experiments, we analyzed the impact of tBRD-2 on gene expression in the testis using RNA of *bam*≫*tbrd-2^RNAi^* testes with testes RNA of *tbrd-2^RNAi^* and *bam-*Gal4 males as controls. Depletion of *tbrd-2* in testes was validated by quantitative PCR (qPCR), western blot analyses, and immunofluorescence microscopy (Fig. 4A–C″). Knockdown of *tbrd-2* led to a significant reduction of *tbrd-2* transcripts compared to control testes (Fig. 4A). Likewise, tBRD-2 was not detected in *bam*≫*tbrd-2^RNAi^* testes in western blots (Fig. 4B) and immunofluorescence analyses (Fig. 4C–C″; Fig. S1). By contrast, transcript and protein levels of *tbrd-1* and *tbrd-3* were not altered in *bam*≫*tbrd-2^RNAi^* testes (Fig. S4A–D″). Further analyses revealed that *bam*≫*tbrd-2^RNAi^* males were sterile (Fig. S5) and exhibited spermatid differentiation defects, e.g. altered Nebenkern formation (Fig. 4D″, arrow) and lack of nuclear elongation (Fig. 4E″; Fig. S1B, arrowheads). In both controls (Fig. 4D,D′), the phase-dark, round Nebenkern was nearly the same size as the nucleus. In *bam*≫*tbrd-2^RNAi^* spermatids, the Nebenkerne seemed to be fused together (Fig. 4D″). Mst77F-positive spermatid nuclei of *bam*-Gal4 (Fig. 4E, arrow) and *tbrd-2^RNAi^* (Fig. 4E′, arrow) were elongated and started to develop the typical needle-like structure of mature sperm nuclei, whereas Mst77F-positive spermatid nuclei of *bam*≫*tbrd-2^RNAi^* did not elongate and remained round (Fig. 4E″, arrow).

**Fig. 5.**
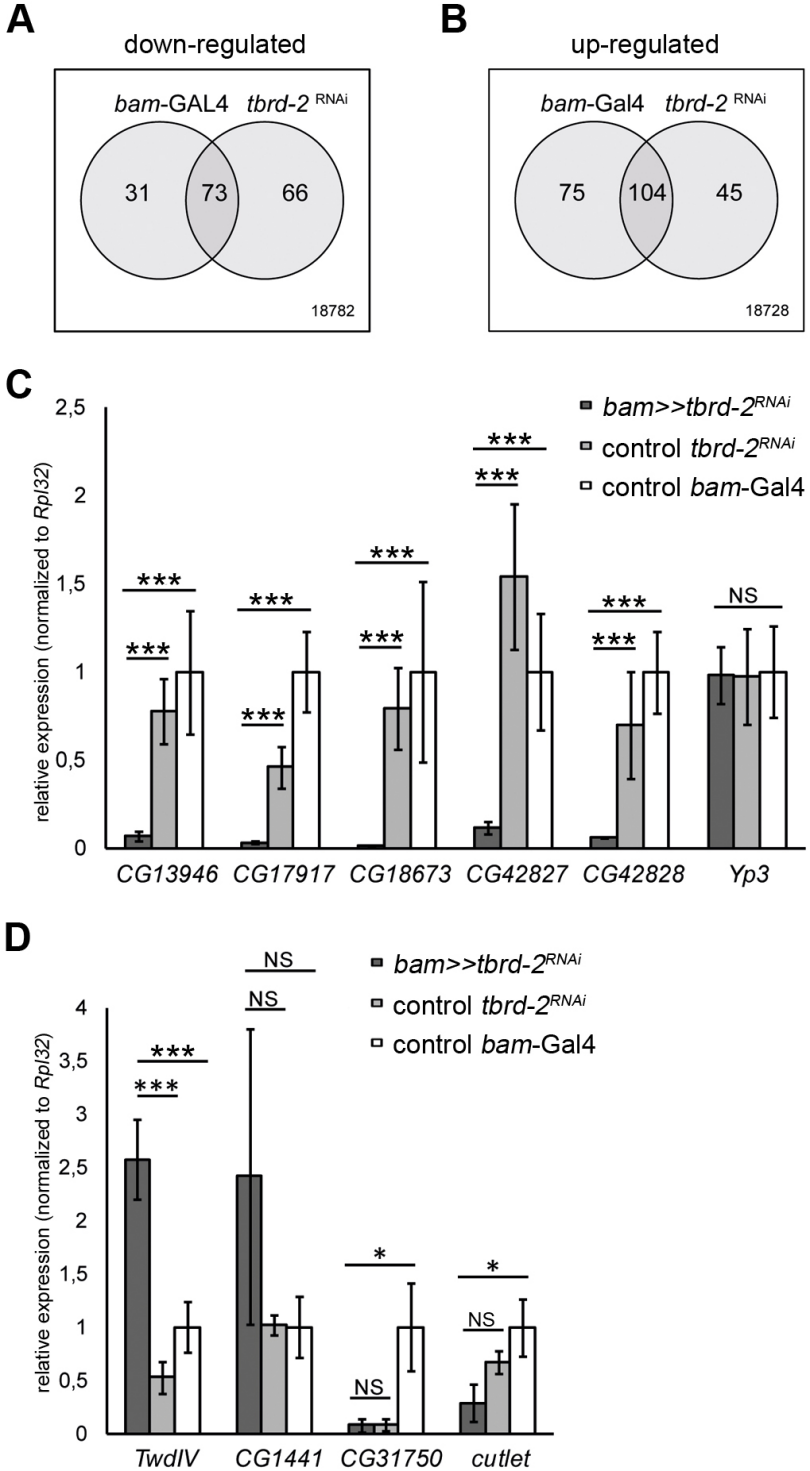
**tBRD-2 is required for gene expression.** (A,B) Venn diagrams depicting the overlap of (A) 73 significantly down-regulated and (B) 104 significantly up-regulated probe sets in *bam*≫*tbrd-2^RNAi^* testes compared to the controls (*bam*-Gal4 and undriven *tbrd-2^RNAi^*). (C,D) qPCR using cDNA from 50 testes pairs of *bam*≫*tbrd-2^RNAi^*, *tbrd-2^RNAi^*, and *bam*-Gal4 testes. (C) Expression of genes *CG13946*, *CG17917*, *CG18673*, *CG42827*, *CG42828* and *Yp3*. (D) Expression of *TwdlV*, *CG1441*, *CG31750* and *cutlet*. The values were normalized to the expression of *Rpl32*. ANOVA with post hoc Tukey's honest significant difference test were used to evaluate statistical significance. *P*-values for significance: *P≤0.05; ***P≤0.001; NS, not significant.

For microarray experiments, Affymetrix *Drosophila* Genome 2.0 arrays were used, and three independent hybridizations per genotype were performed. The expression values for each probe set from the three arrays of the same genotype were averaged, and the log2-fold change between *tbrd-2* knockdown and one of the controls (undriven *tbrd-2^RNAi^* or *bam-*Gal4) was calculated. Knockdown of *tbrd-2* led to a significant down-regulation of 73 probe sets, reflecting 69 protein-coding genes (log2-fold change≤−1; corrPVal ≤0.05) compared to both controls (Fig. 5A); 104 probe sets, reflecting 99 protein-coding genes, were significantly up-regulated (log2-fold change≥+1; corrPVal ≤0.05) (Fig. 5B). As expected, *tbrd-2* was one of the most down-regulated genes in *bam*≫*tbrd-2^RNAi^* testes. In agreement with qPCR results (Fig. S4A), *tbrd-1* and *tbrd-3* were not affected.

In order to identify common target genes of tBRD-2 and tBRD-1, the transcriptomes of *bam*≫*tbrd-2^RNAi^* and *tbrd-1* mutant testes (Theofel et al., 2014) were compared. Among the 69 down-regulated protein coding genes in *bam*≫*tbrd-2^RNAi^*, 38 protein-coding genes were also significantly down-regulated in *tbrd-1* mutants (data not shown). Hence, 55% of the protein-coding genes that were positively regulated by tBRD-2 likewise require tBRD-1. Among the 99 up-regulated protein-coding genes, only 25 were affected in the two transcriptomes (data not shown). In a previous study, we have shown that transcripts of *CG13946*, *CG17917*, *CG18673*, *CG42827*, *CG42828*, and *Yp3* are significantly down-regulated in *tbrd-1* mutant testes, whereas *TwdIV*, *CG1441*, *CG31750* and *cutlet* are significantly up-regulated (Theofel et al., 2014). According to our microarray data presented here, *CG13946*, *CG17917*, *CG18673*, *CG42827*, *CG42828* and *TwdIV* depended on tBRD-2 function, but *Yp3*, *CG1441*, *CG31750*, and *cutlet* did not. Therefore, qPCRs using cDNA of *bam*≫*tbrd-2^RNAi^* and control testes were carried out to validate common and specific tBRD-2 and tBRD-1 target genes (Fig. 5C,D). Indeed, transcript levels of *CG13946*, *CG17917*, *CG18673*, *CG42827*, and *CG42828* were significantly reduced in *bam*≫*tbrd-2^RNAi^* testes compared to controls, but transcript levels of *Yp3* were not (Fig. 5C). Likewise, only transcript levels of *TwdIV* were significantly up-regulated in *bam*≫*tbrd-2^RNAi^* testes (Fig. 5D). Our results demonstrated that tBRD-2 directly or indirectly regulates gene expression in the testis and shares a subset of target genes with tBRD-1.

### tBRD-1, tBRD-2, the tMAC component Aly, the Mediator complex subunit Med22, and the tTAF Sa share a defined set of target genes

**Fig. 6.**
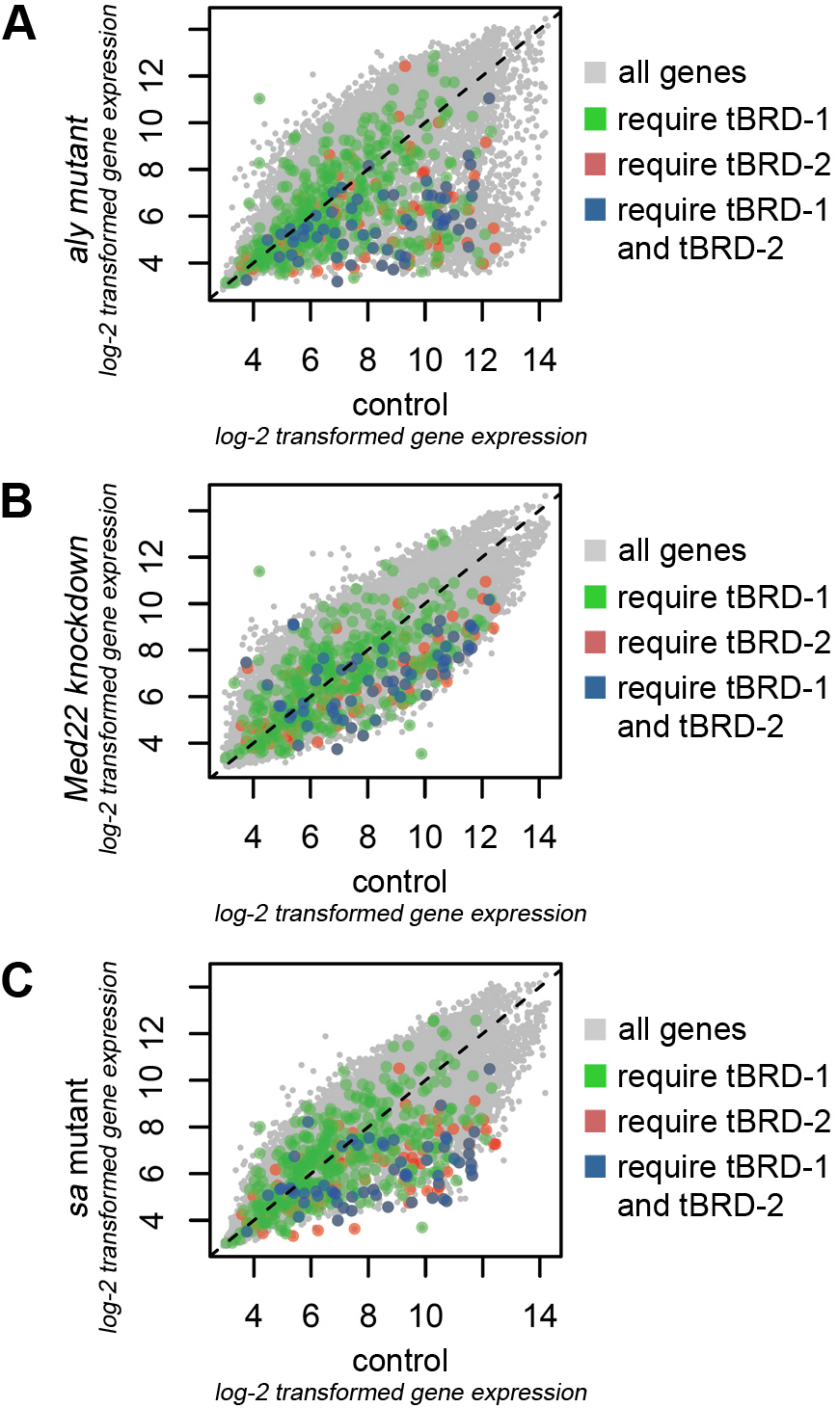
**tBRD-1 and tBRD-2 share a subset of target genes with Aly, Med22, and Sa.** (A–C) Scatter plots depicting transcript levels (log2-transformed gene expression values) in (A) *aly*, (B) *Med22*, and (C) *sa* mutant testes (y-axes) compared to wild-type control testes (*x*-axes). Green and blue dots represent significantly down-regulated genes in *tbrd-1* mutant testes in comparison to control testes. Red and blue dots represent transcripts of genes in *bam*≫*tbrd-2^RNAi^* testes expressed significantly lower than in undriven *tbrd-2^RNAi^*. Blue dots represent transcripts that are affected by both tBRD-1 and tBRD-2.

We compared the transcriptomes of *bam*≫*tbrd-2^RNAi^* (relative to that of undriven *tbrd-2^RNAi^* control testes), *tbrd-1* (Theofel et al., 2014), *aly*, *Med22* and *sa* mutant testes (Lu and Fuller, 2015) (Fig. 6A–C). We focused on the role of tBRD-1 and tBRD-2 in activating transcription. Numerous probe sets significantly down-regulated in *bam*≫*tbrd-2^RNAi^* testes, in *tbrd-1* mutant testes, or in both were likewise down-regulated in *aly* (Fig. 6A), *Med22* (Fig. 6B), and *sa* (Fig. 6C) mutant testes. Of the 447 probe sets that were down-regulated in *tbrd-1* mutants (Tables S1 and S3), 60 were likewise down-regulated in *tbrd-2* knockdown testes (Table S3). Of the 387 probe sets affected in *tbrd-1* but not in *tbrd-2* mutants (Table S1), 71 were likewise down-regulated in all three (*aly*, *Med22*, and *sa*) mutant testes, whereas 231 were unaffected in all of these mutant testes (Table S1). Of the 141 down-regulated probe sets in *tbrd-2* mutants (Tables S2 and S3), 60 were likewise down-regulated in *tbrd-1* mutants (Table S3). Of the 81 probe sets affected in *tbrd-2* but not in *tbrd-1* mutant testes (Table S2), 27 were likewise down-regulated in all three (*aly*, *Med22*, and *sa*) mutant testes, whereas 35 were unaffected. Of the 60 down-regulated probe sets in both *tbrd-1* and *tbrd-2* mutants, 39 were likewise down-regulated in all three (*aly*, *Med22*, and *sa*) mutant testes, whereas 13 were not dependent on Aly, Med22, and Sa function (Table S3). In all three situations (*tbrd-1* with *aly*, *Med22*, and *sa* mutants; *bam*≫*tbrd-2^RNAi^* with *aly*, *Med22*, and *sa* mutants; *tbrd-1*/*bam*≫*tbrd-2^RNAi^* with *aly*, *Med22*, and *sa* mutants) the observed overlap between down-regulated genes was much stronger than expected in a random distribution (*tbrd-1*: hypergeometric *P*<6.6×10^−11^; *bam*≫*tbrd-2^RNAi^*: hypergeometric *P*<9.8×10^−11^; *tbrd-1*/*bam*≫*tbrd-2^RNAi^*: hypergeometric *P*<3.2×10^−28^). By contrast, up-regulated genes only showed minor overlaps that were not significant (Tables S1–S3). In total, 39 probe sets representing 31 protein-coding genes were significantly down-regulated in *bam*≫*tbrd-2^RNAi^*, *tbrd-1*, *aly*, *Med22* and *sa* mutant testes (Table S3). A comparison of this defined set of genes with the *Drosophila* Spermatogenesis Expression Database (http://mnlab.uchicago.edu/sppress/; Vibranovski et al., 2009) revealed that the corresponding transcripts are enriched mainly in post-meiotic male germ cells (Table S4). This led us to postulate that transcription of these genes gives rise to translationally repressed mRNAs coding for spermiogenesis-relevant proteins. In addition, according to FlyAtlas (Chintapalli et al., 2007), most of the transcripts are enriched in the testes (Table S4). Hence, we assume that expression of a precise number of genes, relevant for post-meiotic spermatogenesis, are regulated by all five proteins, namely tBRD-1, tBRD-2, the tMAC component Aly, the Mediator complex subunit Med22, and the tTAF Sa.

### The tMAC component Aly is required for proper sub-cellular localization of tBRD-1 and tBRD-2

**Fig. 7.**
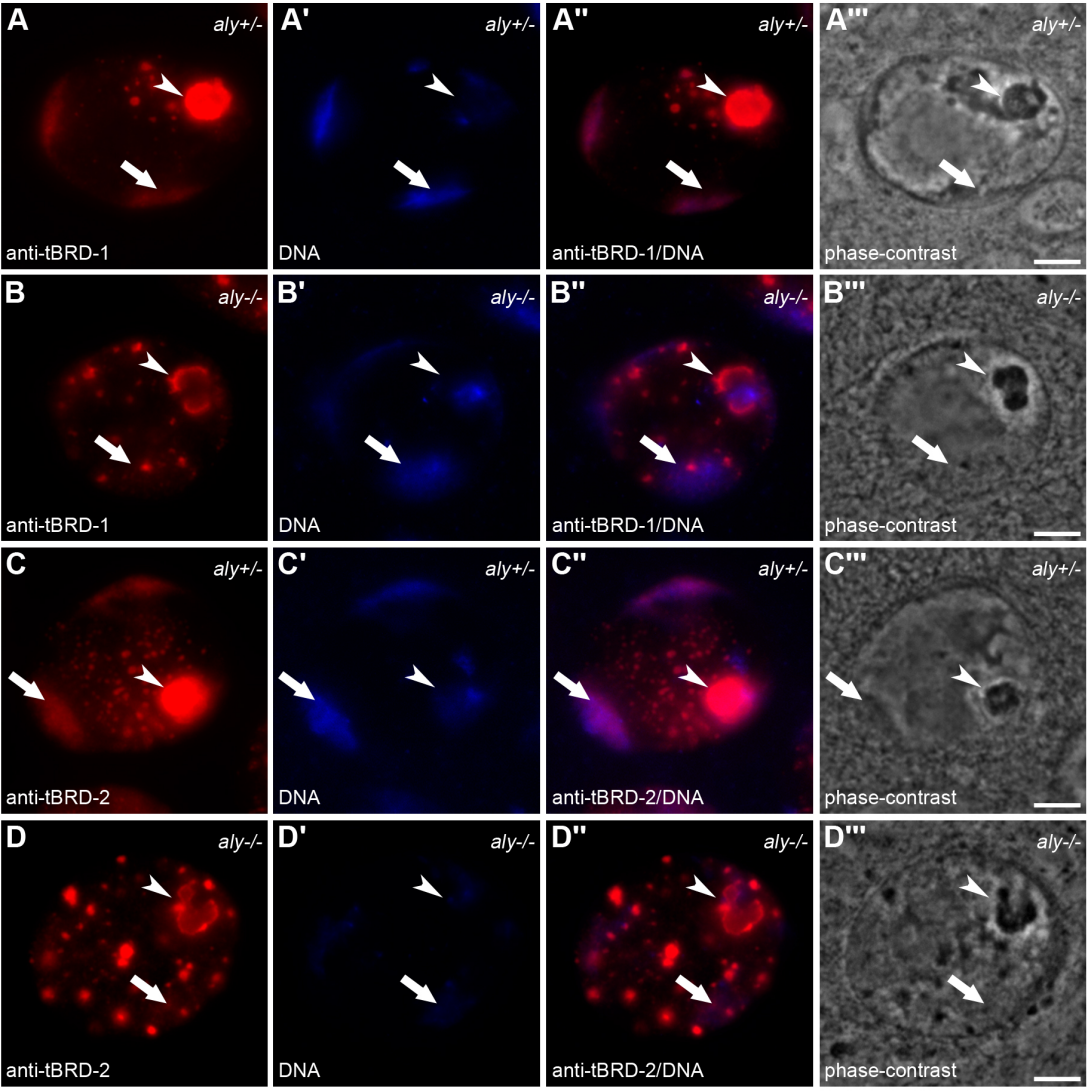
**tBRD-1 and tBRD-2 require Aly function for sub-cellular localization.** (A–D‴) Single primary spermatocyte nuclei of (A–A‴ and C–C‴) heterozygous and (B–B‴ and D–D‴) homozygous *aly* mutants stained with (A–A‴ and B–B‴) anti-tBRD-1 and (C–C‴ and D–D‴) anti-tBRD-2 antibodies. Arrows, chromosomal regions; arrowheads, nucleoli. A,B,C,D, immunofluorescent staining with anti-tBRD-1; A′,B′,C′,D′, Hoechst DNA staining; A″,B″,C″,D″, merged images; A‴,B‴,C‴,D‴, phase-contrast images. Scale bars: 5 µm.

Previously, we have shown that subcellular localization of tBRD-1 and tBRD-2 depends on tTAF function (Leser et al., 2012; Theofel et al., 2014). Here, we analyzed the localization of tBRD-1 and tBRD-2 in heterozygous and homozygous *aly* mutants (Fig. 7). Immunofluorescence staining showed that correct localization of tBRD-1 (Fig. 7A–B″) and tBRD-2 (Fig. 7C–D″) required wild-type Aly function. The localization of tBRD-1 and tBRD-2 to the chromosomal regions was strongly reduced in homozygous *aly^5^* mutant spermatocytes (Fig. 7B,D, arrows). Likewise, the localization of tBRD-1 and tBRD-2 to the nucleoli was clearly reduced (Fig. 7B,D, arrowheads). In addition, tBRD-1- and tBRD-2-positive nuclear speckles were larger and reduced in number in *aly^5^* mutant spermatocytes (Fig. 7B,D).

## DISCUSSION

In *Drosophila*, spermatocytes execute a highly active and strictly regulated transcription program to provide transcripts necessary for post-meiotic spermiogenesis. Transcription of spermiogenesis-relevant genes is based on the cooperation among tTAFs, tMAC components, and Mediator complex components (Beall et al., 2007; Chen et al., 2011; Hiller et al., 2004; Lu and Fuller, 2015). Recently, we have postulated that the testis-specific bromodomain proteins tBRD-1, tBRD-2, and tBRD-3 cooperate with the testis-specific TFIID complex in regulating transcription of a subset of spermiogenesis-relevant genes (Theofel et al., 2014). Here, we uncovered additional potential links between tBRD proteins, Mediator, and tMAC.

### The function of tBRD-1 is essential for proper sub-cellular localization of endogenous tBRD-2

Previously, we have shown that in testes of transgenic flies, endogenous tBRD-1 interacts with tBRD-2-eGFP (Theofel et al., 2014). Here, we further focused on the interaction between tBRD-1 and tBRD-2 and showed that expression of tBRD-1-eGFP can restore sub-cellular localization of tBRD-2 in primary spermatocytes in a *tbrd-1* mutant background. These results indicated that tBRD-1 and tBRD-2 indeed interact in *Drosophila* spermatocytes. The structure of tBRD-1 and tBRD-2 proteins differ from that of classical BET family members in animals, which are mainly characterized by two N-terminal bromodomains and a C-terminal extra-terminal domain consisting of a NET motif and a SEED motif (Florence and Faller, 2001). tBRD-1 contains two bromodomains but no extra-terminal domain, and tBRD-2 contains only one bromodomain but does contain a C-terminal extra-terminal domain (Theofel et al., 2014). The extra-terminal domain has been described as necessary for protein–protein interactions (Florence and Faller, 2001; Matangkasombut et al., 2000; Platt et al., 1999). However, it has been shown that human BRD2 requires the first N-terminal bromodomain for dimerization (Nakamura et al., 2007). More recent results have shown that homodimer and heterodimer formation of BET proteins is mediated by a conserved motif, termed motif B, between the second bromodomain and the extra-terminal domain (Garcia-Gutierrez et al., 2012). We showed in yeast two-hybrid experiments that the C-terminal part of tBRD-1 and the extra-terminal domain of tBRD-2 are essential for interaction of the two proteins. By contrast, homodimer formation of tBRD-1 proteins required the region between the two bromodomains.

Recently, it has been suggested that the interaction of tBRD-1 and tBRD-2 is required for their protein stability (Kimura and Loppin, 2015). However, we did not observe an altered tBRD-1 protein distribution or changes in protein levels in *tbrd-2* knockdown testes compared to controls. tBRD-2 proteins were barely detectable in *tbrd-2* knockdown testes, which allows us to assume that the knockdown was efficient. These results indicated that tBRD-1 does not require tBRD-2 function for protein stability or sub-cellular localization. By contrast, the tBRD-2 signal was strongly reduced in *tbrd-1* mutant spermatocyte nuclei. However, also in this case, we did not observe lower amounts of tBRD-2 protein in *tbrd-1* mutant testes in western blots. Hence, the loss of tBRD-1 seems to affect the sub-cellular localization of tBRD-2. Our results showed that the function of tBRD-1 is required for proper sub-cellular localization of tBRD-2 but not vice versa. In addition, the function of tBRD-1 seems to be dispensable for tBRD-2 protein stability. Whether this dependency is based upon direct interaction of the two proteins still has to be clarified.

### tBRD-1 binds to acetylated histones independently of tBRD-2

Previously, we have shown that an increased acetylation level in spermatocytes enhances the localization of tBRD-1 and tBRD-2 to the chromosomal regions (Leser et al., 2012; Theofel et al., 2014). However, it was unclear whether both proteins directly bind to acetylated histone tails. In the current study, *in vitro* experiments demonstrated that the double bromodomain protein tBRD-1 bind to H3 peptides acetylated at lysines 9 and 14 and to H4 peptides acetylated at lysines 5, 8, and 12. By contrast, tBRD-2 exhibited a higher affinity for non-acetylated histone peptides under the same conditions. Acetylation of N-terminal histone tails of H3 and H4 is a typical feature of transcriptional active chromatin and serves as a binding platform for epigenetic regulators, such as BET proteins (Davie and Candido, 1978; Dhalluin et al., 1999; Hebbes et al., 1988). It has been previously shown that the acetylation marks tested in this study are recognized by BET proteins (Marchand and Caflisch, 2015) and are involved in active gene expression (Morris et al., 2007; Wang et al., 2008). In addition, all tested acetylation marks except those of H3K14ac and H3K36ac were detected in spermatocyte nuclei, which indicated that tBRD-1 recognizes acetylated H3 at lysine 9 and/or 14 and acetylated H4 at lysine 5, 8, and/or 12 also *in vivo*.

In murine round spermatids, acetylated H3 and H4 are enriched at the transcription start sites of spermiogenesis-relevant genes and are recognized by the BET proteins BRD4 and BRDT (Bryant et al., 2015). Recently, it has been suggested that the interaction of tBRD-1 and tBRD-2 allows the two proteins to function together as a single BRDT-like BET protein (Kimura and Loppin, 2015). Therefore, it is conceivable that tBRD-2 requires tBRD-1 for efficient binding to chromatin. However, it is also possible that tBRD-2 recognizes other, not yet tested acetylation marks independently of tBRD-1. As tBRD-1 and tBRD-2 regulate both common and different sets of target genes, both scenarios could occur in spermatocytes. Our data suggest that in *Drosophila*, as in mice, bromodomain proteins act together to efficiently support the activation of spermiogenesis-relevant genes by binding to acetylated lysine residues.

### tBRD-1 and tBRD-2 co-regulate a subset of target genes

Our microarray analyses showed that tBRD-2, like tBRD-1, is involved in gene activation and repression. The comparison of transcriptome data of a *tbrd-1* mutant with that of a *tbrd-2* knockdown clearly indicated that the two bromodomain proteins share a subset of target genes. However, we observed that the expression of some genes was altered in *tbrd-1* mutant testes but not in *tbrd-2* knockdown testes and vice versa, which suggested that some genes are regulated specifically by either tBRD-1 or tBRD-2. In mice, the BET proteins BRDT and BRD4 cooperate to regulate transcription of spermiogenesis-relevant genes, although they can also act independently. Importantly, it has been demonstrated that genes co-bound by BRDT and BRD4 show a higher transcriptional activity than genes bound only by BRD4 or BRDT (Bryant et al., 2015). Further experiments are required to examine whether tBRD-1 and tBRD-2 directly bind to their target genes and whether the binding of both enhances transcription.

### An overlapping set of spermiogenesis-relevant genes is regulated by tBRD-1, tBRD-2, the tMAC complex, Mediator complex, and tTAFs

It has been proposed that the activation of spermiogenesis-relevant genes in *Drosophila* spermatocytes requires the sequential action of the tMAC complex, Mediator complex, and testis-specific TFIID (tTFIID) complex (Chen et al., 2011; Lu and Fuller, 2015). The tMAC component Topi interacts with the Mediator component Med22, but no direct interaction has been observed between Mediator and tTFIID components. However, when *Med22* is knocked down, the tTAF Sa fails to localize to chromatin, which suggests that tTAFs depend on Mediator to be recruited to chromatin or stabilized there (Lu and Fuller, 2015). Previously, we have shown that the proper localization of tBRD-1 and tBRD-2 depends on tTAF function (Leser et al., 2012; Theofel et al., 2014). In addition, we have demonstrated that tBRD-1 and the tTAF Sa share a subset of target genes (Theofel et al., 2014). In our current study, immunofluorescence analyses revealed a dramatically reduced localization of tBRD-1 and tBRD-2 to chromosomal regions in homozygous *aly* mutant spermatocytes. We hypothesized that also tBRD-1 and tBRD-2 are involved in the gene regulatory cascade in spermatocytes recently proposed by Lu and Fuller (2015). Therefore, we compared our *tbrd-1* and *tbrd-2* mutant transcriptome data with that of *sa*, *aly*, and *med22* mutants (Lu and Fuller, 2015; Theofel et al., 2014). Indeed, a defined subset of 31 genes were regulated by all five factors. The transcripts of most of these genes are enriched in the testes and accumulate in post-meiotic germ cells (Chintapalli et al., 2007; Vibranovski et al., 2009), which suggests that these transcripts are among the translationally repressed mRNAs required for spermatid differentiation. In contrast to Sa, Aly, and Med22, tBRD-1 and tBRD-2 are involved in the regulation of only a small number of genes. Expression of known tTAF-, tMAC-, and Mediator-dependent spermiogenesis-relevant genes, e.g., *fzo*, *janB*, *gdl* and *CG9173*, is not affected in *tbrd-1* and *tbrd-2* mutants. Nevertheless, our data showed that tBRD-1, tBRD-2, Sa, Aly, and Med22 regulate a common set of genes. We hypothesize that tBRD-1 and tBRD-2 act at the end of a gene regulatory cascade involving tMAC, Mediator, and tTAF functions to regulate expression of spermiogenesis-relevant genes.

## MATERIALS AND METHODS

### Fly strains and RNAi experiments

Flies were maintained under standard conditions at 25°C. *w^1118^* was used as the wild-type strain. To knockdown *tbrd-2,* homozygous males of the RNAi line v37671 (Vienna *Drosophila* Resource Center; Dietzl et al., 2007) were crossed against virgins of *bam*-Gal4/*bam*-Gal4; Sp/CyO; *bam*-Gal4-VP16/MKRS (Caporilli et al., 2013; Chen and McKearin, 2003). *bam*≫*tbrd-2^RNAi^* males analyzed in this study carried two copies of *bam-*Gal4 (on chromosomes X and III) and one copy of *tbrd-2^RNAi^* (on chromosome III). Undriven *tbrd-2^RNAi^* control males carried one copy of *tbrd-2^RNAi^*, and *bam-*Gal4 control males carried two copies of *bam-*Gal4 (on chromosomes X and III). RNAi crossings (including control flies) were maintained at 30°C. The transgenic line *tbrd-1-eGFP* and the *tbrd-1* mutant strain *tbrd-1^1^* have been recently described (Leser et al., 2012). *aly^5^* mutants were kindly provided by Helen White-Cooper (Cardiff University, School of Biosciences).

### Fertility tests

Batches of 20 flies were tested for fertility. Adult males were crossed individually against two wild-type virgin females in separate vials at 25°C. After 6 days, the parental generation was removed. The number of offspring was counted after two weeks.

### Immunofluorescence stainings

Testes squash preparations were immunofluorescently stained essentially as described in Hime et al. (1996) and Rathke et al. (2007). A peptide antibody was raised against tBRD-2 (amino acids 436–457) in rabbit (Pineda-Antibody-Service; http://www.pineda-abservice.de). The affinity-purified antibody was used at a dilution of 1:1000. Anti-tBRD-1 was applied at a dilution of 1:5000 (Leser et al., 2012). Anti-tBRD-3 (Theofel et al., 2014) and anti-Mst77F (Rathke et al., 2010) were used at a dilution of 1:1000. Anti-histone (Millipore; clone F152.C25.WJJ) was used at a dilution of 1:1200. To detect acetylated histones, the following antibodies were used: anti-H3K9ac (Sigma-Aldrich, H9286; 1:500), anti-H3K14ac (Active Motif, Carlsbad, CA, USA, 39698; 1:500), anti-H3K18ac (Active Motif, 39756; 1:500), anti-H3K23ac (Active Motif, 39132; 1:600), anti-H3K27ac (Active Motif, 39136; 1:500) and anti-H3K36ac (Active Motif, 39380; 1:250). H4K5ac, H4K8ac, and H4K12ac were detected using the acetyl-histone H4 antibody set (17-211) from Millipore. DNA was visualized via Hoechst staining. As secondary antibodies, Cy3-conjugated anti-rabbit (Dianova; 1:100), Cy2-conjugated anti-rabbit (Dianova; 1:100), and Cy5-conjugated anti-mouse (Dianova; 1:100) were used. Immunofluorescence stainings were examined using a Zeiss microscope (AxioPlan2). Figures were designed using Adobe Photoshop CS2.

### Western blot experiments

Western blot experiments were performed as recently described (Leser et al., 2012). For each protein extract, 20 testes of heterozygous or homozygous *tbrd-1^1^* mutants or *bam*≫*tbrd-2^RNAi^*, undriven *tbrd-2^RNAi^*, or *bam-*Gal4 were used. Anti-tBRD-1, anti-tBRD-2, and anti-Pan-Actin (Cell Signaling Technologies, 4968) were used at a dilution of 1:1000.

### Yeast two-hybrid assays

Yeast two-hybrid interaction tests were performed using the Matchmaker™ GAL4 Two-Hybrid System 3 from Clontech according to the manufacturer's manual. tBRD-1 and tBRD-2 full-length yeast constructs are described in Theofel et al. (2014). Mutated *tbrd-1* and *tbrd2* ORFs were PCR amplified using specific primers with linked restriction sites (Table S5) and ligated into pGADT7 (bait vector) and pGBKT7 (prey vector). Translational start and stop codons were introduced via the specific primers. To amplify *tbrd-1ΔC* (base pairs 1–492) the primer pair *tbrd-1ΔC-fw/tbrd-1ΔC-rv* was used. *tbrd-1ΔC2* (base pairs 1–1227) was amplified using *tbrd-1ΔC-fw/tbrd-1ΔC2-rv*. *tbrd-1ΔN* (base pairs 493–1542) was amplified using *tbrd-1ΔN-fw/tbrd-1ΔN-rv*. *tbrd-1Δ* (base pairs 382–1008) was amplified using *tbrd-1Δ-fw/tbrd-1Δ-rv*. To amplify *tbrd-2ΔC* (base pairs 1–1062), the primer pair *tbrd-2ΔC-fw/tbrd-2ΔC-rv* was used. *tbrd-2-ΔN* (base pairs 1060–2025) was amplified using *tbrd-2ΔN-fw/tbrd-2ΔN-rv*. The *tbrd-2ΔBD* consists of two parts. Base pairs 1–150 were amplified using the primer pair *tbrd-2ΔBD-fw1/tbrd-2ΔBD-rv1*, and base pairs 358–2025 were amplified using the primer pair *tbrd-2ΔBD-fw2/tbrd-2ΔBD-rv2*. The two PCR products were ligated together into the TOPO-TA vector (Life Technologies) using XmaI and XhoI. The constructed final *tbrd-2ΔBD* containing the required restriction sites to ligate the PCR product into pGADT7 or pGBKT7 was amplified using the primers *tbrd-2-Y2H-NdeI-fw* and *tbrd-2-Y2H-EcoRI-rv*. The first part of *tbrd-2ΔNET* (base pairs 1–1086) was amplified using *tbrd-2ΔNET-fw1/tbrd-2ΔNET-rv1*; the second part (base pairs 1330–2025) was amplified using *tbrd-2ΔNET-fw2/tbrd-2ΔNET-rv2*. The two parts were ligated together into the TOPO-TA vector (Life Technologies) using XmaI and XhoI. The constructed final *tbrd-2ΔNET* was amplified using *tbrd-2-Y2H-NdeI-fw*/*tbrd-2-Y2H-EcoRI-rv*. To amplify *tbrd-2ΔSEED* (base pairs 1–1740) the primer pair *tbrd-2ΔSEED-fw/tbrd-2ΔSEED-rv* was used. *tbrd-2ΔNETΔSEED* (base pairs 1–1086 and 1330–1740) was amplified using *tbrd-2ΔNET* as a template and the primer pair *tbrd-2-Y2H-NdeI-fw/tbrd-2-ΔSEED-rv*.

### RNA isolation and microarray experiments

Total RNA was isolated from *bam*≫*tbrd-2^RNAi^*, undriven *tbrd-2^RNAi^*, and *bam-*Gal4 testes using TRIzol (Invitrogen). RNA quality was monitored using the Agilent Bioanalyser 2100 with the RNA 6000 Nano kit. Gene expression was analyzed using Affymetrix *Drosophila* Genome 2.0 arrays according to the manufacturer's recommendations. For each array, independent RNA from whole testes pooled from 25 animals was used. Three independent replicates were prepared for each experimental condition. The data were analyzed in the R statistical environment using BioConductor packages (Huber et al., 2015). Scanned data were parsed as CEL files into R using the /affy/ package (Gautier et al., 2004). Expression estimates were extracted using RMA normalization with the /rma/ function. Differentially expressed genes were identified using Limma (Ritchie et al., 2015). Genes with log2 (fold expression change) >1 or<−1 and an adjusted *P*-value <0.05 were selected as significantly up- or down-regulated, respectively.

For comparison with previously published data (Lu and Fuller, 2015), we downloaded CEL files from the GEO repository (GSE74784) and processed them as described above to obtain log2-transformed expression measures. To make the different data sets comparable, initial RMA was applied to the complete data set.

The microarray data were deposited at the NCBI gene expression omnibus (GEO) under the accession number GSE81019.

### Quantitative real-time PCR

Total RNA from 100 *bam*≫*tbrd-2^RNAi^* testes, undriven *tbrd-2^RNAi^* testes, and *bam-*Gal4 testes was extracted using TRIzol (Invitrogen). RNA was treated with RQ1 RNase-Free DNase (Promega). For cDNA synthesis, 1 µg DNase-digested RNA and the Transcriptor First Strand cDNA Synthesis Kit (Roche) were used. qPCR reactions contained 7.5 μl iTaq™ Universal SYBR^®^ Green Supermix (Bio-Rad), 5.2 μl ddH_2_O, 2 μl diluted cDNA, 0.3 μl (10 μM) gene-specific primer 1, and 0.3 μl (10 μM) gene-specific primer 2. qPCR (three technical replicates) was performed with a Sybrgreen platform on a Bio-Rad CFX Cycler. Data were analyzed using Bio-Rad CFX Manager™ software. Values were normalized to the mRNA expression level of *Rpl32*. Differences between groups were determined with analyses of variance. Delta Ct values were analyzed for ANOVA using the aov function of R. For the differences between individual groups post hoc tests were calculated by Tukey's honest significant difference test (TukeyHSD function). Two groups were compared using one-way ANOVA. The corresponding *P*-values indicated in the figures are *P≤0.05, **P≤0.01, and ***P≤0.001.

Primers are given in Table S6.

### Expression and purification of recombinant tBRD-1 and tBRD-2

*tbrd-1* and *tbrd-2* cDNAs were FLAG tagged at the C-terminus by PCR using specific primers and ligated into the baculovirus transfer expression vector pVL1392. Transfection of Sf9 cells, recombinant baculovirus production, and recombinant protein expression and purification essentially followed the methods described in Brehm et al. (2000).

### Peptide pull-down experiments

H3 and H4 peptides were synthesized (PSL Peptide Specialty Laboratories) and coupled to SulfoLink™ coupling resin (Thermo Scientific) according to the manufacturer's instructions. One microgram of each peptide was added to 1 µl beads; 2.5 µl of the coupled beads were mixed with 17.5 µl uncoupled beads and washed in pull-down buffer (25 mM Tris-HCl, pH 8.0, 150 mM NaCl, 2 mM EDTA, 0.5% NP-40, 1 mM DTT, proteinase inhibitors) for 5 min twice. After blocking for 1 h at 4°C in blocking buffer [1 mg/ml BSA, 1% fish skin gelatin (Sigma) in pull-down buffer], beads were incubated with 0.25 µg recombinant proteins for 2 h at 4°C. Beads were washed four times in pull-down buffer. Bound proteins were analyzed by SDS-PAGE and western blotting using tBRD-1- and tBRD-2-specific antibodies; 20% of the input was loaded on the gel.
